# The new WHO guideline for control and elimination of human schistosomiasis: implications for the Schistosomiasis Elimination Programme in Nigeria

**DOI:** 10.1186/s40249-022-01034-3

**Published:** 2022-10-24

**Authors:** Akinola Stephen Oluwole, Uwem Friday Ekpo, Obiageli Josephine Nebe, Nse Michael Akpan, Solomon Monday Jacob, Uche Veronica Amazigo, John Russell Stothard

**Affiliations:** 1 Department of Pure and Applied Zoology, Federal University of Agriculture, Abeokuta, Ogun State Nigeria; 2 Neglected Tropical Disease Control Division, Department of Public Health, Federal Ministry of Health, Abuja, Nigeria; 3 Pan-African Community Initiative on Education and Health (PACIEH), Enugu, Nigeria; 4 Department of Tropical Disease Biology, Liverpool School of Tropical Medicine, Liverpool, L3 5QA UK

## Abstract

**Supplementary Information:**

The online version contains supplementary material available at 10.1186/s40249-022-01034-3.

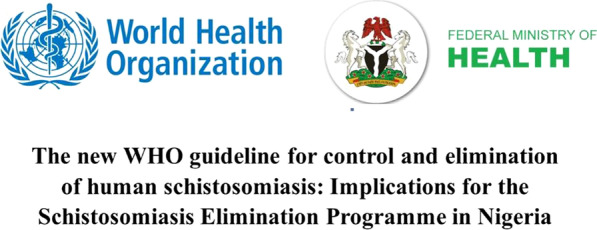

## Background

With some 134,073,166 people living in endemic communities at risk of infection [1], Nigeria is the most endemic country in Africa and requires preventive chemotherapy (PC) for a total of 26.3 million persons [2]. The National Schistosomiasis Elimination Programme (NSCHEP), with the support of international partners, has been implementing PC in Nigeria since 2009 and most recently will need to revise its current strategy (Additional file 1). For example, the new World Health Organization (WHO) guideline has six key recommendations that will dramatically change the implementation of schistosomiasis elimination in endemic countries [3]. However, its impact and programmatic implications will vary from country to country, hence the need for a country-specific analysis. This article discusses these recommendations with specific reference to the challenges and opportunities in Nigeria. We summarise the key pointers in Additional file 1: Box 1 against the six recommendations of the WHO 2022 guideline.

### Implications for the Schistosomiasis Elimination Programme in Nigeria

# 1. Additional platforms for delivering praziquantel must be identified aside from the school-based PC. Increased allocation of praziquantel tablets is required for PC in Nigeria. To this end, a spatially explicit map of schistosomiasis prevalence with the estimated number of persons requiring praziquantel treatment per community is imperative to guide the implementation of this recommendation. The georeferencing of all schistosomiasis’s endemic communities and validation of Expanded Special Project for Elimination of Neglected Tropical Diseases ward level prevalence data for Nigeria should be prioritized. Ward level prevalence maps and population data to estimate treatment needs from > 2 years are required to guide implementation. NSCHEP implementation guideline also needs to be revised to include PC of preschool children, pregnant women after the first trimester and lactating mothers. A challenge will be accessing the required donated praziquantel for Nigeria in the face of global scarcity.

# 2. Stopping PC requires careful consideration, particularly for communities where PC is seen as government largesse. However, a decision to reduce the frequency of PC requires close epidemiological monitoring to ensure there is no rebound in prevalence. Implementation of test-and-treat will require a point-of-care (POC) rapid diagnostic kit for both types of schistosomiasis and praziquantel at a nearby health facility. This requires critical consideration of cost, which is currently outside PC activities and budget. POC kits will need to be imported, with the training of health workers on how to use the kits. Such training may be extended to the local pharmacy in hard-to-reach communities where there are no health facilities.

# 3 NSCHEP and partners should/will identify endemic communities (≥ 10% prevalence) for biannual treatment with persistently high prevalence despite rounds of PC. The decision to implement biannual treatment will require prior conduct of impact assessment surveys to identify hot-spot communities, which has cost implications for funding partners. Furthermore, regular safeguarding against any future erosion of community acceptance of offered treatments should be secured.

# 4 The integration of the schistosomiasis control programme into the primary health care system to provide access to all age groups at proven or suspected risk, is a crucial step in response to the global call for schistosomiasis elimination. The feasibility of this was recently demonstrated by a pilot COUNTDOWN female genital schistosomiasis (FGS) health system study where sixty-six women with symptoms of FGS were able to access praziquantel treatment outside PC in Ogun State, Nigeria [4]. Availability of praziquantel at the health facilities will require innovative management of the drug supply chain and reverse logistics by programme managers and implementers. This transition needs careful forward planning and stakeholder co-ordination to be robust.

# 5. There is a need for a collaborative effort and sharing of NTD and WASH data among stakeholders. The National Schistosomiasis Technical Working Group, NTDs WASH sub-committee and State Advisory Committee on Neglected Tropical Disease (SACON), comprising representatives from the Academia, WASH, National Orientation Agency, Primary Health Care Board, Media, Ministry of Education, and implementers should work collaboratively. SACON should be empowered and supported to develop an integrated NTD and WASH database for monitoring WASH and NTD activities. We also advocate the use of helminthiases as bio-indicators of WASH sector activity impact. NSCHEP should adopt research-proven innovative behavioural change and health education tools for sensitisation among people living in the endemic communities [5]. Efforts should be made to increase human resources and capacity in vector snail control, through appropriate training, following WHO manuals on molluscicides.

#6. NSCHEP should leverage existing collaboration with schistosomiasis experts within academic and research institutions in Nigeria to develop MSc/PhD research projects at the request of Federal Ministry of Health (FMoH) for impact assessment, monitoring, and to establish the interruption of transmission in post-MDA communities. The collaboration will reduce the burden of FMoH on funding these verification activities, as such studies could establish and secure a research grant platform for its execution. A notable gap in current applied research is the lack of an appraisal of zoonotic schistosomiasis, alongside its contribution to the transmission of human schistosomiasis in Nigeria.

## Conclusions

NSCHEP and stakeholders must meet and issue new policy documents to domesticate the implementation of the new WHO guideline with support from local WHO offices WHO-AFRO/Nigeria. A critical dimension to consider is the raising of sufficient domestic funding, to fill potential gaps likely to emerge in the implementation of the new recommendations. This is particular apt in light of dwindling external funding for the national NTD programme.

## Supplementary Information

**Additional file 1:** Opportunities and Challenges of the new WHO recommendations.

## Data Availability

Not applicable.

## Abbreviations

FGS: Female genital schistosomiasis
FMoH: Federal Ministry of Health
NTDs: Neglected tropical diseases
NSCHEP: The National Schistosomiasis Elimination Programme
PC: Preventive chemotherapy
POC: Point-of-care
PZQ: Praziquantel
SAC: School-aged children
SACON: State Advisory Committee on Neglected Tropical Disease
WASH: Water, sanitation and hygiene
WHO: World Health Organization
