# Supplementary material for: The new WHO guideline for control and elimination of human schistosomiasis: implications for the Schistosomiasis Elimination Programme in Nigeria
Source: Infect Dis Poverty. 2022 Oct 24;11:111. doi: 10.1186/s40249-022-01034-3 (PMC9590168; doi:10.1186/s40249-022-01034-3)
Supplement: Supplementary file 1 — Additional file 1: Opportunities and Challenges of the new WHO recommendations. [file 40249_2022_1034_MOESM1_ESM.docx]

**Supplementary file 1**

**The new WHO guideline for control and elimination of human schistosomiasis: Implications for the Schistosomiasis Elimination Programme in Nigeria**

**Additional Background**

The NTD unit of the Federal Ministry of Health recently changed the programme title from Schistosomiasis Control Programme to Schistosomiasis Elimination Programme to depict the new target of the programme, schistosomiasis elimination is now being implemented at the sub-district (ward) level, with full disaggregation of endemicity data from the Local Government Area (LGA) or District level to the sub-district (ward). Nigeria has a total of 9684 wards, of which 6363 are endemics for schistosomiasis; 252 wards have high endemicity (≥ 50%), 2,981wards have moderate endemicity (10–49.9%) and 3130 wards have low endemicity (1–9.9%). Currently, 39,540,516 School-Aged Children (SAC) and 9,442,358 adults require preventive chemotherapy based on the old WHO guidelines (WHO, 2006).

**Implications for the Schistosomiasis Elimination Programme in Nigeria**

Highlighted below are key opportunities and challenges of implementing the five key recommendations for the Schistosomiasis Elimination programme in Nigeria.

***Recommendation 1: In endemic communities with prevalence of Schistosoma spp. infection ≥ 10%, WHO recommends annual preventive chemotherapy with a single dose of praziquantel at ≥ 75% treatment coverage in all age groups from 2 years old, including adults, pregnant women after the first trimester and lactating women, to control schistosomiasis morbidity and advance towards eliminating the disease as a public health problem.***

***Opportunities***

This recommendation from WHO is critical and has the potential of reducing schistosomiasis transmission and ensure that everyone affected or at risk of schistosomiasis (from age 2 and above) get access to treatment early enough to reduce onset of morbidity resulting from long term exposure to the disease. A good example is Female Genital schistosomiasis which has become a global concern. Also, the recommendation of ≥10% prevalence as benchmark for mass drug administration implies the need to conduct precision mapping for Mass Drug Administration even with non-endemic wards and update the list of eligible endemic communities. In addition, the number of persons eligible for treatment and estimate the number of praziquantel tablets require review in the light of the inclusion of the new target group as contained in the new WHO Guideline

**Challenges**

The National Schistosomiasis Elimination programme in Nigeria needs to consider a change in the treatment strategies from the school-based approach to an innovative approach that will ensure that no one is left behind. The challenges of treating preschool-aged children (2 years to 4 years) who are not yet in schools and who need parental consent to take PQZ is there. Besides, there are no resources to access pediatric praziquantel for now. Additional financial and logistical implications would be required to successful implement recommendation #1, which may be a challenge in view of the dwindling funding for NTD programme. Also, quantity of medicine requirement will increase which may be a challenge for the company producing the medicine, since the cost of increasing production may increase

**Recommendation 2: *In endemic communities with prevalence of Schistosoma spp. infection < 10%, WHO suggests one of two approaches based on programmatic objectives and resources: (i) where there has been a programme of regular preventive chemotherapy, to continue the intervention at the same or reduced frequency towards interruption of transmission; or (ii) where there has not been a programme of regular preventive chemotherapy, to use a clinical approach of test-and-treat, instead of preventive chemotherapy targeting a population.***

***Opportunity***

This recommendation will help to identify communities for stopping mass drug administration and transit to test and treat approach, or if continued at a reduce frequency. This will go a long way to keep prevalence below the threshold of < 10%, in the face of absence of complimentary intervention such as WASH and behavioral change and Health Education. Opportunity for assessment of elimination as a public health problem will only occurred in such communities.

**Challenges**

Successful implementation of the option one of this recommendation require that sentinel site survey or mid-term evaluation must be thoughtfully plan and budgeted for as part of the routine programme implementation strategy. This of course will mean extra funding for programme implementation which may be challenging in the face of a dwindling funding available from donor organizations coupled with poor counterpart funding from Government.

Also, there will be need for availability of praziquantel at the health facility beside MDA to implement the test and treat approach recommended by WHO. This is currently a challenge faced by the Schistosomiasis elimination programme, simply because the structure of the supply chain management does not permit praziquantel tablet to be available beyond the period of the MDA campaign. In view of this recommendation, the National Schistosomiasis Elimination Programme will need to review the supply chain management structure to allow for retainment of the drug beyond the MDA period. There will be also the need for the national programme to procure and make available Point-of-Care (POC) test kits for both types of schistosomiasis at health facilities, which have cost and supply chain implication, which need to be address.

***Recommendation 3: In endemic communities with prevalence of Schistosoma* *spp. infection ≥ 10% that demonstrate lack of an appropriate response to annual preventive chemotherapy, despite adequate treatment coverage (≥ 75%), WHO suggests consideration of biannual (twice yearly) instead of annual preventive chemotherapy.***

***Opportunity***

The implication is that the National Schistosomiasis Elimination Programme and its implementing partner need to demonstrate the existence of hot-spots communities first. This requires the programme to have achieved ≥ 75% treatment coverage in these communities consistently for many rounds and impact assessment survey to be qualify for biannual treatment instead of annual preventive chemotherapy. Implementing this recommendation will give opportunity to understand and identify the factors contributing to persistence transmission of schistosomiasis, which can be addressed by stakeholders.

**Challenges**

Implementation of this recommendation, require addition funds for conducting impact assessment surveys as well as implementing the second round of MDA which require funding by implementing partners

**Recommendation 4: *WHO recommends that health facilities provide access to treatment with praziquantel to control morbidity due to schistosomiasis in all infected individuals regardless of age, including infected pregnant excluding the first trimester, lactating women, and pre-SAC aged < 2 years? The decision to administer treatment in children under 2 years of age should be based on testing and clinical judgement.***

**Opportunity**

Implementing the above recommendation, will require the National Schistosomiasis Control Programme to develop policy to ensure that praziquantel are always made available at the primary health. This will give opportunity for all time access to praziquantel treatment for those at risk, especially those who are unable to access it during the MDA period or due to programme policy.

**Challenges**

All round availability of drugs at the health facilities will depend strongly on the efficiency of the drug supply chain management by programme managers and implementers at the state, national and the supporting partners. This is currently a challenge in Nigeria has there have been report of late arrival of drugs affecting programme delivery. It is more critical considering the short shelf-life of praziquantel (2 years). It is important that the drugs get to the end users before it expired, using an efficient drug supply chain management.

**Recommendation 5: *WHO recommends WASH interventions, environmental interventions (water engineering and focal snail control with molluscicides) and behavioral change interventions as essential measures to help reduce transmission of Schistosoma spp. in endemic areas.***

**Opportunity**

Implementing this recommendation will help in the reduction of transmission by ensuring the Schistosoma egg does not get into water bodies, and the elimination of the snail intermediate host. This will require the participation of relevant agencies such Ministries of Health, Environment, Water Resources, and Information. The policy makers at National Schistosomiasis Elimination programme and at the State level will need to pay advocacy visits to these ministries mentioned above and request them to be part of the stakeholders in the planning and implementation of the schistosomiasis elimination programme and gain their cooperation and commitment towards elimination of NTD.

**Challenges**

The challenge of implementing this recommendation is the lack of capacity in snail vector control in Nigeria. Lack of political will to target and prioritize schistosomiasis endemic communities for WASH intervention.

**Box 1.** Summary of implications and opportunities for Nigeria for each of the six recommendations

|  |
| --- |
| ***Recommendation 1- In endemic communities with the prevalence of Schistosoma spp. infection ≥ 10%, WHO recommends annual preventive chemotherapy with a single dose of praziquantel at ≥ 75% treatment coverage in all age groups from 2 years old, including adults, pregnant women after the first trimester and lactating women, to control schistosomiasis morbidity and advance towards eliminating the disease as a public health problem***  ***#1.*** The school-based strategy which targets only school-aged children will no longer suffice. Hence, the need for a new or adapted strategy or additional platform for reaching pre-school-aged children, pregnant women and lactating women during PC.  A higher resolution spatially explicit map of schistosomiasis prevalence with an estimated number of persons requiring praziquantel treatment per community is needed.   1. These maps must be accessible to implementers at ward levels. 2. Building the capacity of frontline health workers to create, and query such maps is important/necessary   Develop modalities to treat special at-risk groups (> 2 years old, pregnant women after the first trimester and lactating women) in health facilities.  A review of national schistosomiasis elimination policy documents to capture the new realities. |
| ***Recommendation 2 - In endemic communities with the prevalence of Schistosoma* *spp. infection < 10%, WHO suggests one of two approaches based on programmatic objectives and resources:***  ***#2.*** There is a need for the programme to review and decide to either stop treatment/or continue at a reduced frequency. For Option 2, the resource to carry out the test and treat in nearby health facilities will be a challenge. How to provide the test kits? What will happen in a community where there are no health facilities nearby? What training is required for the test and treat options? Are partners willing to carry this burden, other than stopping PC and deleting < 10% communities from their PC database? There may be a need to consider the training of local pharmacies in test and treat where there is no health facility to address Option 2. |
| ***Recommendation 3*** ***-In endemic communities with prevalence of Schistosoma spp. infection ≥ 10% that demonstrate lack of an appropriate response to annual preventive chemotherapy, despite adequate treatment coverage (≥ 75%), WHO suggests consideration of biannual (twice yearly) instead of annual preventive chemotherapy.***  ***#3.*** It requires identifying communities with hyper-endemicity levels of schistosomiasis where prevalence has remained high ranging between 60 and 80% despite several rounds of treatment (require impact assessment). Biannual treatment to replace the annual preventive chemotherapy in these communities should be incorporated into the NSCHEP yearly activity. it is also important to note that this will require additional funds for implementing the second round of Mass administration which initially are not budgeted for by most implementing partners. |
| ***Recommendation 4 - WHO recommends that health facilities provide access to treatment with praziquantel to control morbidity due to schistosomiasis in all infected individuals regardless of age, including infected pregnant excluding the first trimester, lactating women, and pre-SAC aged < 2 years? The decision to administer treatment in children under 2 years of age should be based on testing and clinical judgement.***  #4. Integration of schistosomiasis elimination programme into the primary health care system to increase access to these groups which are not currently captured by the PC programme. In addition, all-year-round availability of praziquantel/paediatric praziquantel at the health facilities will depend strongly on the efficiency of the drug supply chain management by programme managers and implementers at the state, national and supporting partners. The FMoH will engage the necessary partners. |
| ***Recommendation 5 - WHO recommends WASH interventions, environmental interventions (water engineering and focal snail control with molluscicides) and behavioural change interventions as essential measures to help reduce transmission of* Schistosoma *spp. in endemic areas.***  #5. It requires a collaborative effort between the Ministry of Health, Ministry of Environment, the ministry of water resources, the National Orientation Agency, and the Ministry of information. The NTD unit of the Federal Ministry of Health, Nigeria already set up a structure in all States where NTD is endemic to foster this collaboration, this is known as State Advisory Committee on Neglected Tropical Disease. Programme implementers at the state level should leverage this committee to drive home the importance of WASH towards the elimination of NTD. Some document on the strategy of operation such as WASH and NTDs has been released by WHO to help with this. Each of the stakeholders should be aware of their role and be committed to it.  Focal application of molluscicides may be necessary for some endemic areas |
| ***Recommendation 6 -* In communities approaching the interruption of transmission (defined as having no autochthonous human cases reported for 5 consecutive years), WHO suggests a verification framework**  #6. NSCHP should leverage existing collaboration with schistosomiasis experts within academic and research institutions in Nigeria to address some of the implementation challenges, it will be interesting to have studies that help address zoonotic schistosomiasis as there is no information on this in Nigeria. FMoH should strengthen and empower the State Advisory Committee on NTDs (SACON) to provide oversight and elimination activities. |
